# Seed Biofortification and Phytic Acid Reduction: A Conflict of Interest for the Plant?

**DOI:** 10.3390/plants4040728

**Published:** 2015-11-20

**Authors:** Francesca Sparvoli, Eleonora Cominelli

**Affiliations:** Institute of Agricultural Biology and Biotechnology, CNR, Via Bassini 15, 20133 Milan, Italy; E-Mail: cominelli@ibba.cnr.it

**Keywords:** gene regulation, inositol phosphates, mineral deficiency, phytate, signal transduction

## Abstract

Most of the phosphorus in seeds is accumulated in the form of phytic acid (*myo*-inositol-1,2,3,4,5,6-hexa*kis*phosphate, InsP_6_). This molecule is a strong chelator of cations important for nutrition, such as iron, zinc, magnesium, and calcium. For this reason, InsP_6_ is considered an antinutritional factor. In recent years, efforts to biofortify seeds through the generation of low phytic acid (*lpa*) mutants have been noteworthy. Moreover, genes involved in the biosynthesis and accumulation of this molecule have been isolated and characterized in different species. Beyond its role in phosphorus storage, phytic acid is a very important signaling molecule involved in different regulatory processes during plant development and responses to different stimuli. Consequently, many *lpa* mutants show different negative pleitotropic effects. The strength of these pleiotropic effects depends on the specific mutated gene, possible functional redundancy, the nature of the mutation, and the spatio-temporal expression of the gene. Breeding programs or transgenic approaches aimed at development of new *lpa* mutants must take into consideration these different aspects in order to maximize the utility of these mutants.

## 1. Introduction

Phytic acid (*myo*-inositol-1,2,3,4,5,6-hexa*kis*phosphate, InsP_6_) is a ubiquitous component of eukaryotic cells which, together with its metabolism, plays a number of regulatory roles [1]. In plants, phytic acid is the most abundant form of phosphorus occurring in seeds (up to 85% of total phosphorus and with amounts even 1000 fold higher than those detected in vegetative tissues) and other plant tissues and organs such as pollen, roots, tubers and turions. During seed development, the synthesis of phytic acid is coordinated with that of other seed storage compounds. Accumulation significantly increases after the “cell division phase”, reaching a plateau at the end of the “cell expansion phase” [2,3]. In mature seeds, phytate is organized into spherical inclusions called globoids, which are in turn found within protein bodies. Phytate deposits are also observed to occur transiently in various tissues and subcellular compartments during grain development [4,5,6,7]. Depending upon the species, the amount and distribution of phytic acid in different parts of the seed can be quite variable, with the most striking differences found in cereal grains. In the case of barley, wheat and rice, a large amount (80%) of phytic acid is stored in the aleurone and bran (maternal teguments) and only a limited amount accumulates in the embryo. The distribution of phytate is opposite in maize seeds, where 80% of phytate accumulates in the embryo and scutellum [8]. In the case of legume seeds, such as common bean, more than 95% of seed phytic acid is accumulated in the cotyledons [9], while in the model species *Arabidopsis*, phytic acid is mostly stored in the embryo [5]. During germination, in order to support seedling growth, phytic acid is then degraded by phytase enzymes to remobilize the phosphorus stored as phytate salts [10].

Due to its chemical structure (highly negatively charged at physiological pH), phytic acid easily precipitates in the form of phytate salts, binding important mineral cations such as iron, zinc, potassium, calcium, and magnesium. Monogastric animals, including humans, lack phytases in their digestive tract and fail to process the phytates present in seeds, thus phytic acid is poorly digested and decreases the nutritional value of the seeds by limiting phosphorus and mineral bioavailability. Poor mineral bioavailability, due to high molar ratios between phytic acid and mineral cations, is ascribed as one of the most important causes of mineral deficiencies (mainly iron and zinc) in populations whose diet is largely based on staple crops [11,12]. On the other hand, InsP_6_ is largely excreted by nonruminants. A common practice to provide for an animal’s nutritional requirement for phosphorus (P) has been the supplementation of feed with nutrient P. However, this procedure increases P concentration in manure, leading to P accumulation in soils, and the consequent risk of P pollution in runoff water [13]. To obviate these problems, a solution is the development of low phytic acid (*lpa*) crop seeds [14].

The availability and management of P in agriculture is a challenging global problem: reserves of rock P are non-renewable and enhanced uptake and utilization of P would be of value for agricultural production in P-deficient environments throughout the world. It would also contribute to the long-term goal of sustainable and environmentally friendly agricultural production [14,15]. The total P accumulated in seed crops of major grains and legumes represents in sum more than 50% of the total P fertilizer used annually worldwide [16]. Therefore, reducing seed total P, together with a reduction of phytate content, might also contribute to these goals [17]. This has been achieved at least for the barley *lpa1-1* mutant, in which seeds show a decrease both in phytic acid and total P [18].

The biological functions of phytic acid and the identification of genetic resources and strategies useful in engineering high-yielding, stress-tolerant low-phytate germplasm have been reviewed by a number of authors [10,11,14,17]. However, in most cases, a strong emphasis was placed on the agronomic aspects of the topic, with only modest efforts to integrate these aspects with the emerging knowledge of the regulatory role of phytic acid and inositol metabolism. The aim of this review is to summarize the most recent results in the literature about relevant aspects of the phytic acid pathway and *lpa* mutants. Moreover, we discuss some pleiotropic effects of *lpa* mutants with respect to the reported roles, important for cell signaling and plant processes, of phytic acid and key enzymes and metabolites of this complex pathway.

## 2. Biosynthetic Pathways

**Figure 1:**
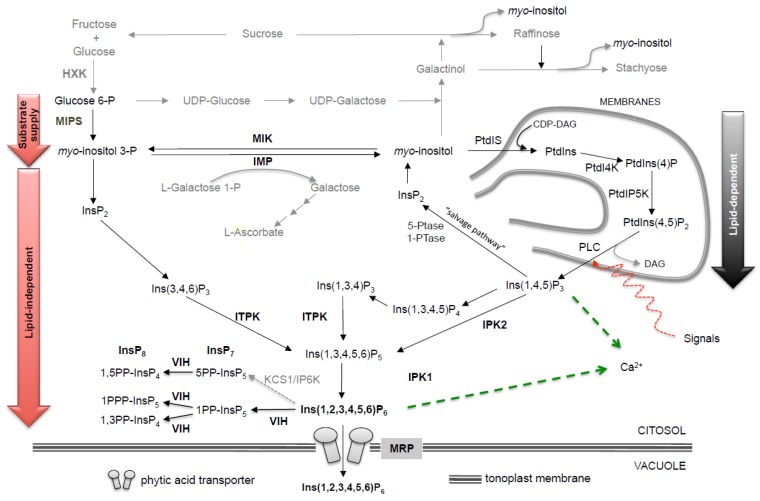
Schematic representation of phytic acid biosynthetic pathway (black) and *myo*-inositol derived pathways for ascorbic acid and raffinose-type oligosaccharides (grey). The substrate supply, lipid independent (red) and lipid dependent (dark grey) sub-pathways for myo-inositol-1,2,3,4,5,6-hexakisphosphate (InsP_6_) synthesis are indicated. MIPS, *myo*-inositol-3-phosphate synthase; IMP, bifunctional enzyme: *myo*-inositol-phosphate monophosphatase and galactose-1-phosphate phosphatase; MIOX, *myo*-inositol monooxygenase; MIK, *myo*-inositol kinase; IPK2, inositol 1,4,5-tris-phosphate kinase; ITPK, inositol 1,3,4-triphosphate 5/6-kinase; IPK1, inositol 1,3,4,5,6 penta*kis*phosphate 2-kinase; PtdIS, phosphatidyl inositol phosphate synthase; PtdI4K, phosphatidyl inositol 4-kinase; PtdIP5K, phosphatidyl inositol 4-phospate 5-kinase; PtdIns,phosphatidyl inositol; PtdInsP_1_, phosphatidyl inositol monophosphate; PtdInsP_2_, phosphatidyl inositol biphosphate; PLC, phospholipase C; MRP, multidrug-resistance-associated protein ATP-binding cassette; HXK, hexokinase; VIH, diphosphoinositol pentakisphosphate kinase; KCS1, inositol hexakisphosphate kinase. Dotted grey line indicates a second route for InsP_7_ synthesis, existing in eukaryotes but not found in plants.

In plants, it is now generally accepted that InsP_6_ biosynthesis occurs through two different routes: a “lipid-dependent” pathway, which is ubiquitous in eukaryotic cells, and a “lipid-independent” pathway (Figure 1). The first operates in all plant tissues, while the second appears to predominanate in seeds. Phytic acid biosynthesis needs the *de novo* production of *myo*-inositol (hereafter referred to as “Ins”) through a highly conserved reaction, shared by all living organisms, in which the enzyme d-*myo*-inositol 3-phosphate synthase (MIPS) converts d-glucose-6-phosphate to *myo*-inositol 3-phosphate (Ins(3)P_1_) (Figure 1). *Myo*-inositol 3-phosphate is then dephosphorylated to free Ins by a specific Mg^2+^-dependent inositol monophosphate phosphatase (IMP). Interestingly, the IMP enzyme has a dual activity: besides Ins(3)P_1_, it also hydrolyzes l-galactose 1-phosphate (l-Gal 1-P), a precursor of ascorbic acid synthesis [19,20]. The reaction catalyzed by IMP can be reversed by the action of *myo*-inositol kinase (MIK). Since Ins(3)P1 is produced directly from glucose 6-phosphate by MIPS, it is not clear exactly why MIK activity is important for InsP_6_ biosynthesis, although the importance of MIK in seed InsP_6_ metabolism has been demonstrated by a number of mutations in the *MIK* gene [21,22,23]. A possible explanation is that MIK could provide more substrate diversity for the generation of inositol bisphosphate to feed the lipid-independent pathway, since it is able to produce multiple inositol monophosphates [21].

The main difference between the “lipid-dependent” and “lipid-independent” routes is the way inositol tri-phosphates (InsP_3_) are generated. In the “lipid-dependent” pathway, Ins is converted to phosphatidylinositol (PtdIns) by a phosphatidylinositol synthase (PtdIS). Next, the headgroup of PtdIns is sequentially phosphorylated by phosphatidylinositol kinases to produce PtdIns(4,5)P_2_. This molecule is the substrate of a PtdIns-specific phospholipase C activity that releases Ins(1,4,5)P_3_, a molecule central to signal transduction [24].

The so-called “lipid-independent” pathway is entirely independent from inositol lipid synthesis and consists of sequential phosphorylation of the Ins ring to InsP_6_, through the action of a number of specific inositol phosphate kinases. As already mentioned, the first phosphorylation step, consisting in the conversion of Ins to InsP_1_, is catalyzed by *myo*-inositol kinase (MIK). The production of InsP_2_ from InsP_1_ requires a monophosphate kinase. A good candidate for this step is a homolog of 2-phosphoglycerate kinase (2-PGK), which catalyzes the production of 2,3-6 bisphosphoglycerate from 2-phosphoglycerate in archaea [14]. In rice, a mutation in this gene (*OsPGK1*) generates an *lpa* phenotype, while overexpression increases seed InsP_6_ content, suggesting that *OsPGK1* is a key gene for InsP_6_ synthesis, being involved in (probably the rate-limiting) step from InsP_1_ to InsP_2_ [22,25,26]. Further phosphorylation steps, required to convert InsP_3_ into the more phosphorylated InsP_4_, InsP_5_ and InsP_6_, involve at least three types of inositol kinases: (i) the evolutionarily conserved inositol phosphate kinase 2, also known as inositol polyphosphate multikinase (IPK2/IPMK), which is a dual 6-/3-kinase; (ii) the inositol 1,3,4-trisphosphate 5-/6-kinase (ITPK) belonging to the family of ATP-grasp fold proteins; and (iii) the inositol polyphosphate 2-kinase (IPK1), which specifically phosphorylates InsPs in the 2-position.

Once synthesized, phytic acid is stored as globoids inside the storage vacuoles where it is actively transported by a specific InsP_6_ transporter, a multidrug-resistance-associated protein (MRP), belonging to the ATP-binding cassette (ABC) family [27]. The involvement of all the above-mentioned proteins in InsP_6_ synthesis and accumulation is supported by direct enzyme isolation and biochemical characterization and/or by the isolation of corresponding *lpa* mutants [19,28,29,30,31,32,33,34] (Table S1).

### 2.1. Genomic Organization and Regulation of Phytic Acid Pathway Genes

Genes involved in phytic acid biosynthesis and transport have been characterized in *Arabidopsis thaliana* and crop plants such as rice, wheat, soybean and common bean, through forward (through the screening for *lpa* mutations) and reverse genetics [35,36,37,38,39]. In this section we will describe the main classes of genes coding for enzymes of the pathway, *MIPS*, *IMP*, *MIK*, *2-PGK*, *IPK2*, *ITK* and *IPK1*, and *MRP* genes coding for InsP_6_ transporters. Most of these are members of small gene families, with the exception of *MIK*, which is typically encoded by a single copy locus [35]. However, some differences among species have been reported concerning the presence of single or multiple loci for each gene function. Moreover, the tissue specificity of expression can vary among members of a gene family, and may indicate redundancy in vegetative or seed tissues. All these aspects contribute significantly to the establishment of possible *lpa* mutant phenotypes.

#### 2.1.1. MIPS

The *MIPS* gene is a prime example of variable loci numbers reported across species. In fact, in the barley genome, the presence of only one *MIPS* gene has been described, while two are present in rice and common bean, and several are found in *Arabidopsis*, maize, and soybean [36,37,40,41,42,43,44]. One of two *MIPS* genes in rice and in common bean, and one of four in soybean were highly expressed in developing seeds [36,37,40] and their down-regulation in rice and soybean, through mutation or RNAi, caused an *lpa* phenotype in seeds [43,45,46,47]. However, a systematic study performed on mutants isolated in *Arabidopsis* showed that no single mutant in three *MIPS* genes induces an *lpa* phenotype in seeds, suggesting a redundant role for these genes [35]. Interestingly, a reduction in InsP_6_ in leaves was reported for *atmips1* and *atmips2* mutant plants. Moreover, *atmips2* showed increased susceptibility to viruses, fungi and bacteria, while *atips1* did not, suggesting a different degree of redundancy between the two genes in vegetative *versus* seed tissues [48]. Promoter-*GUS* fusion analyses of the three *Arabidopsis* genes showed overlapping activity of *AtMIPS1*, *AtMIPS2* and *AtMIPS3* promoters at the globular stage of seed development [44]. At later stages, strong GUS staining was present in the entire seed only for *AtMIPS1*, while *AtMIPS2* and *AtMIPS3* promoter activity was limited to the seed coat and funiculus [44]. These data suggest an essential role of *myo*-inositol synthesis at early stages of embryo development, but not at later stages, when the synthesis of *myo*-inositol mediated by AtMIPS1 alone seems to be sufficient. However, as the complete knock out of *AtMIPS1* caused neither an *lpa* phenotype in the seed [35] nor embryo abnormalities, under normal (non stressed) conditions as observed in double and triple mutant (embryo lethality in this last case) [44], it can be deduced that the *myo*-inositol synthesized in the endosperm and maternal tissues by AtMIPS2 and AtMIPS3 can be transported into the embryo [44]. Immunolocalization studies, with an antibody against the three AtMIPS isoforms, showed that AtMIPS proteins appear to be specifically located within the endosperm cytosol both at torpedo stage and in mature seed, but not in the embryo, where phytic acid is accumulated [49]. These data on protein localization do not exactly correlate with the expression of *AtMIPS1* [44], suggesting that a post-trascriptional regulation of *AtMIPS* expression is present in the seed. Moreover, the presence of AtMIPS proteins in the endosperm, a seed compartment where InsP_6_ is not accumulated, implies a complex interaction between endosperm and embryo during the synthesis and subsequent accumulation of InsP_6_ during *Arabidopsis* seed development [49]. In contrast, *RINO1* (the rice seed *MIPS* gene) transcript was first detected at the apex of embryos of developing rice seeds [42]. Consequently, its expression colocalizes with InsP_6_-containing globoids within the scutellum and aleurone layers [4]. In soybean, *GmMIPS1* expression was first detected in maternal tissue, and then in the embryo and cotyledons [50]. In both monocots and dicots, *MIPS* expression starts very early during seed development, a few days before the beginning of accumulation of phytic acid, and then decreases [36,37].

#### 2.1.2. *Myo*-Inositol Reversible Dephosphsorylation: IMP and MIK

*IMP* genes form a small family including *IMP* genes, similar to mammalian ones, and *IMPL* (*IMP-like*) genes, coding for chloroplastic proteins more similar to prokaryotic *IMP* genes [19,36]. A different number of loci coding for IMP enzymes has been reported in different species: one *IMP* gene in barley and in common bean [37,51], one *IMP* and one *IMPL* gene in rice [36], one *IMP* and two *IMPL* genes in *Arabidopsis* [19], and three *IMP* genes in tomato [52]. The three *Arabidopsis* genes are expressed in several tissues with *AtIMP* showing the highest levels of expression, except in seeds, where *AtIMPL1* is predominant [53]. Interestingly, a time course analysis of seed development showed IMP and IMPL gene expression correlated with that of *SAL1/FRY*, which codes for *myo*-inositol polyphosphate 1-phosphatase. This enzyme is involved in the *myo*-inositol “salvage pathway” and SAL1/FRY expression only partially overlaps the expression of *AtMIPS* genes. This expression pattern suggested that the salvage pathway may also be involved in *myo*-inositol synthesis during seed development [54]. More recently, it was shown that AtIMPL2 functions in the histidine biosynthetic pathway while AtIMP and AtIMPL1 catalyze the hydrolysis of inositol- and galactose-phosphates in *Arabidopsis* vegetative tissues [53]. During common bean seed development, *PvIMP* expression is similar to that of *MIPS*, being very high at early stages, and then declining to undetectable levels before the start of phytic acid accumulation [37].

To date, one *MIK* gene has been identified in plant genomes [21,23,35,37]. This gene is expressed at high levels during seed development, as shown in *Arabidopsis* and common bean [35,37].

#### 2.1.3. PGK

Genes coding for 2-PGK have been characterized only in rice and *Arabidopsis* [22,25,26]. In rice, *OsLpa1* (*OsPGK1*) is expressed in shoot, root, and panicle [23]. In *Arabidopsis*, two genes of this family were described, *At5g60760* and *At3g45090*. They are present in different organs, however only *At5g60760* is highly expressed during silique development and is ncessary for InsP_6_ synthesis [22]. Among the different *Arabidopsis* genes involved in phytic acid synthesis in siliques, *At5g60760* together with *AtMIK* is the most highly expressed [35].

#### 2.1.4. IPK2

IPK2 kinase is specific for the lipid-dependent pathway, which is not the major route to phytic acid in the seed [14]. However, a low phytate seed phenotype (reduction of phytic acid content by 35%–48%) in the *Arabidopsis* *atipk2*β mutant indicates the lipid-dependent pathway is active in the seed [55]. Despite similar expression in vegetative and reproductive tissues, mutation of *AtIPK2*α has no effect on levels of phytic acid in the seed, suggesting independent roles for the two genes in seed development [35]. *AtIPK2*α plays also a role in pollen germination and root growth [56], while *AtIPK2*β is involved in axillary shoot branching through the auxin signaling pathway [57] and its expression in tobacco leads to improved tolerance to abiotic stresses [58]. Consistent with the role of IPK2 in later steps of phytic acid synthesis, *IPK2* transcripts, when assessed during seed development, showed the highest accumulation later than *MIPS* and *IMP*.

#### 2.1.5. ITPK

ITPK proteins cluster into three phylogenetic subgroups: α, β and γ [34,36,39]. Six different ITPKs have been described in rice [36], four in *Arabidopsis* [32,59,60], soybean [39] and wheat [38], at least three in common bean [37] and one in maize [61,62]. Among the six rice *ITPKs* genes, *OsITP5/6K-4* (α subgroup) and *OsITP5/6K-6* (γ), showed seed-specific expression, with *OsITP5/6K-4* transcript being particularly abundant in the aleurone and *OsITP5/6K-6* in the embryo [36]. For *OsITP5/6K-6* (*Os09g34300*), a knock out mutant with *lpa* phenotype was recently described [63]. In *Arabidopsis*, the *AtITPK1* belongs to the α subgroup, *AtITPK2* and *AtITPK3* to the β and *AtITPK4* to the γ. *AtITPK* genes are expressed in different tissues, with none specifically expressed in siliques [32,64]. *AtITPK1* and *AtITPK4* *lpa* mutants have been isolated, indicating that these two genes are not redundant [35]. An explanation for their non-redundant function is that AtITPK4 lacks inositol 3,4,5,6-tetrakisphosphate 1-kinase activity, characteristic of other AtITPKs, but instead shows inositol 1,4,5,6-tetrakisphosphate and inositol 1,3,4,5-tetrakisphosphate isomerase activity [32]. Soybean *GmITPK3*, showed higher expression in early stages of seed development compared to the other *GmITPKs* [39], and belongs to the β subgroup. The *ITPK* maize gene, *ZmIPK* (α subgroup), showed embryo-specific expression, and the mutation affecting this gene causes an *lpa* phenotype [62]. *ITPK* genes are generally expressed at similar levels during seed development with a decrease in expression at later phases for some members of the family [36,37,38].

#### 2.1.6. IPK1

A single *IPK1* gene was described in several crop plants. The rice *OsIPK1* shows the highest expression in the aleurone between 7 and 10 days after anthesis [36]; common bean *PvIPK1* expression does not appear to be significantly regulated during seed development [37]; and wheat *TaIPK1* doubles its expression during seed development [38]. Two *IPK1* genes were described in maize and in *Arabidopsis*. The maize *ZmIPK1A* gene is expressed in a range of tissues including immature ears, seeds at 12 DAF, middle-stage endosperm and maturing embryos, while *ZmIPK1B* is expressed in roots [33]; the two *Arabidopsis* *IPK1* genes are expressed in different tissues, with only one expressed in siliques that causes an 83% reduction in seed phytate level when knocked down [31,55]. Three *IPK1* genes were identified in soybean and only one is highly expressed in the seed [65].

#### 2.1.7. MRP

The first gene coding for an ABCC-type InsP_6_ transporter was mapped to the maize *ZmMRP4* locus [66]. The functional characterization of this gene family was performed with the *Arabidopsis* homolog AtMRP5 (also referred to as AtABCC5) [67], previously characterized for its involvement in regulating stomatal movements and drought tolerance [68,69]. Phylogenetic analyses of MRP type transporters indicate that InsP_6_ transporters are represented as single copy genes [70,71,72], as described for *Arabidopsis* *AtMRP5*, maize *ZmMRP4* and rice *OsMRP5* [66,67,73]. *ZmMRP4* and *OsMRP5* are expressed in different organs including seeds [66,73]. *AtMRP5*-promoter fusion to a *GUS* reporter gene showed staining mainly in vascular tissues and in guard cells, with no staining in seeds [74]. However, from publicly available microarray data, it is clear that *AtMRP5* is expressed at different stages of seed development [75]. It has been recently shown that soybean and common bean, two closely related legume species, bear a paralog copy (PvMRP2 and GmMRP13) of the genes characterized for their role in phytic acid accumulation (PvMRP1 and GmMRP3 and GmMRP19) [76,77]. In common bean *PvMRP1* and *PvMRP2* are expressed in almost all tissues at similar levels, with the exception of developing seeds, where *PvMRP1* is expressed at consistently higher levels than *PvMRP2* (unpublished resultsA similar behavior is found for the corresponding soybean genes [78].

## 3. Low Phytic Acid (*lpa*) Mutants

Several *lpa* mutants have been isolated in important grain crops, such as barley, maize, rice, wheat, soybean, common bean and pea (Table S1). *lpa* mutations can be grouped into three classes, depending on the affected step of the biosynthetic pathway or mode of compartmentation/transport: (i) mutations involved the first steps of the pathway (from glucose 6-P to *myo*-inositol 3-phosphate), commonly indicated as “supply pathway”; (ii) mutations perturbing the end of the pathway (from *myo*-inositol 3-phosphate to InsP_6_); and (iii) mutations affecting tissue compartmentation of InsP_6_ and/or its transport and storage to the vacuole (MRP transporter) (Figure 1). Mutants belonging to the first and the third classes are generally characterized by decreased InsP_6_ levels accompanied by a molar equivalent increase in inorganic P_i_, but not by accumulation of lower InsP_s_ (inositol phosphates with up to five phosphate residues), a characteristic specific of the second class of mutants (Table 1).

The similarities between the first and third class of mutants triggered some confusion in the initial characterization of some *lpa* mutants in the InsP_6_ transporter genes (*MRP*). The first hypothesis was that they were affected in the *MIPS* gene [61,79]. In fact, a reduced expression of the *ZmMIPS1S* gene was reported in the maize *lpa1* mutant. However, mapping of the maize *lpa1* locus led to the identification of a defective *ZmMRP4* [66]. Available maize genomic data helped to identify that the *ZmMIPS1S* and the *ZmMRP4* genes map very closely on chromosome 1S, thus explaining the incorrect association of the mutation to the *ZmMIPS1S* gene [61,66,79,80]. Phylogenetic studies identified the AtABCC5/AtMRP5, a high affinity InsP_6_ ATP-binding cassette transporter, as the closest *Arabidopsis* homologue of ZmMRP4 [67]. Later, *lpa* mutations already isolated in rice, soybean and common bean [81,82,83] were shown to affect genes orthologous to ZmMRP4/AtMRP5 [73,76,77,84,85].

**Table 1:** Classification of *lpa* mutations on the basis of the affected function in the pathway for phytic acid synthesis and accumulation.

Class	Gene Function	Effects on the Pathway
Type 1	MIPS	Decrease in phytic acid accompanied by a molar increase in free phosphate
	MIK	
	IMP	
Type 2	2PGK	Decrease in phytic acid accompanied by a low increase in free phosphate and increased content of lower inositol phosphates (InsPs)
	IPK2	
	ITPK	
	IPK1	
Type 3	MRP	Decrease in phytic acid accompanied by a molar increase in free phosphate and/or decrease in phytic acid in specific seed tissues
	Putative sulfate transporter (sultr3;3)	

### Metabolic and Phenotypic Traits of Low Phytic Acid Mutants

To ensure mineral bioavailabity, the reduction of the phytate:mineral cation molar ratio is very important, thus a consistent phytic acid reduction is highly desirable in lpa mutants. Unfortunately, negative agronomic traits, such as low germination rates, reduced seed development and weight, and stunted vegetative growth, have been frequently reported for many *lpa* mutants, making them of limited value to breeders [61,86,87,88,89]. For many crops, obtaining *lpa* mutants with no or very limited pleiotropic agronomic defects still remains a challenging goal, although a few well performing *lpa* mutants have been reported [90,91,92]. In barley, at least one *lpa* mutant (*Hvlpa1-1*) has been shown to be equivalent to or even better than its wild type parent. In addition, some *lpa* mutations do not severely affect the yields, especially in non-stressful production environments, thus suggesting that at least some *lpa* mutants show potential for use in breeding [90,93] and a number of *lpa* varieties have been registered [94,95].

The number and strength of pleiotropic effects on InsP_6_ biosynthesis in *lpa* mutants are the result of differences in copy number (functional redundancy) and the spatio-temporal expression of genes and their respective products (protein and metabolite localization). In addition, an increasing number of studies describe tight integration of InsP_6_ metabolic and signaling pathways. The existence of complex metabolic crosstalk among enzyme activities and the type and abundance of different InsPs in a differentiated cell types and tissues may result in unexpected phenotypes (reviewed by [96,97,98,99,100]).

**Figure 2:**
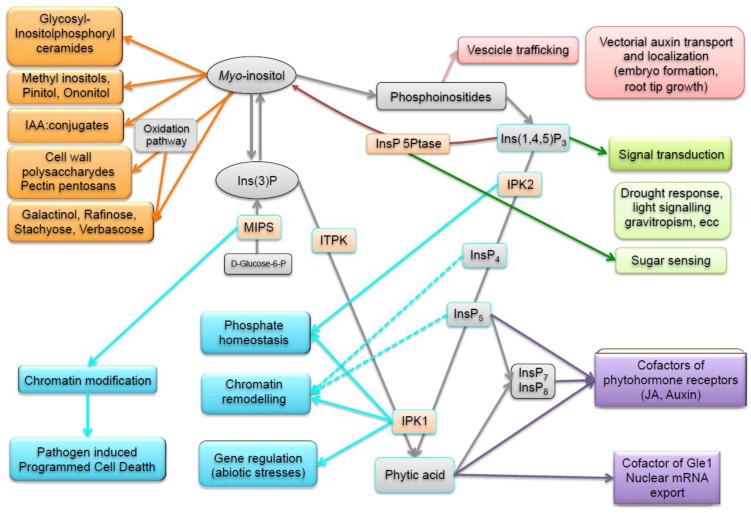
Scheme showing and summarizing the diverse roles of inositol metabolism and phytic acid pathway in compatible pathways (orange), signal transduction (green), membrane biogenesis and trafficking (red), gene regulation (light blue), and as cofactors of regulatory proteins (violet). Light blue boxed enzymes and metabolites have been found localized both in the nucleus and in the cytosol.

The most severe pleiotropic defects have been found in *lpa* mutants carrying defective *MIPS* or *MRP* genes, as well as in mutants in other genes that result in phytic acid reductions of more than 70% (Table S1 and references therein). These negative effects on agronomic performance and seed viability are not surprising, given that most of the *lpa* mutations affect *myo*-inositol and other metabolites that are important for normal development. Since MIPS activity is the only source of the *myo*-inositol ring, and since *myo*-inositol is an essential cellular metabolite important to numerous pathways and functions (Figure 2), perturbing MIPS expression may prove deleterious, if not lethal, even if MIPS suppression is specifically targeted to the seed. For example, *mips* soybean mutants (L33 and Gm-lpa-TW75-1), with 50% phytic acid decrease, showed reduced field emergence, especially when seeds were produced in a subtropical environment (the so-called “seed-source” effect), while high seed abortion and 95% phytic acid reduction occur in transgenic soybean plants with an almost complete suppression of the *MIPS* gene by RNAi [45,86,101]. Defects in embryogenesis and embryo lethality have been reported for *Arabidopsis* *mips* mutants [102,103]. Maize and rice *lpa* mutants, with defects in the *MRP* gene, have very similar phenotypes, and the severity of the phenotype (seedling or embryo death) correlates with the extent of phytic acid reductions [61,73,88,104,105]. Surprisingly, no embryo defects neither seed lethality/abortion have been reported for known *mrp* mutants of dicot species (soybean, common bean and *Arabidopsis*), although phytic acid is significantly reduced in their seeds [67,82,85]. Most likely, tissue and cellular compartmentation of the phytic acid pathway significantly contributes to the effects on seed development caused by perturbations due to *lpa* mutations [14,50].

Altered *myo*-inositol contents have been reported in many *lpa* mutants. As expected, *mips* and *mik* mutants have seed *myo*-inositol levels lower and higher than wild type, respectively (Table S1 and references therein). The soybean mutant *Gm-lpa-ZC-2*, harboring a non-functional IPK1 protein, has increased *myo*-inositol levels [65]. Contrasting data are reported in *mrp* mutants of maize and common bean. In these mutants *MIPS* gene expression is downregulated, thus lower seed *myo*-inositol content would be expected. This occurs in the bean *lpa1* and the maize *lpa1-241* mutants, however higher seed *myo*-inositol has been reported in the maize *lpa1-1* mutant [66,76,106]. An inverse relationship between *MIPS* expression and seed *myo*-inositol content is also observed in two allelic *mrp* mutations of rice and in three barley *lpa* mutants (*lpa2-1*, *lpa3-3* and *M955*), for which the molecular defects are still unknown [81,107]. Changes in *myo*-inositol content also affect the synthesis of derived metabolites, such as galactinol and raffinosaccharides (Table S1) [43,76,107,108,109,110]. Seed *myo*-inositol content has also been suggested to correlate with response to ABA during seed germination [19,76,111]. The *Arabidopsis* and common bean *lpa* (*mrp*) mutants have opposite *myo*-inositol seed contents and also show opposite responses to ABA during germination, which is strongly inhibited in common bean *lpa1* (*Pvmrp1*) mutant, while it is unaffected in the *Arabidopsis* *mrp5* seeds [68,76]. Ins(1,4,5)P_3_(InsP_3_) levels are critical for ABA response in germinating seeds, as ABA hypersensitivity is accompanied by an increase in InsP_3_ catabolism [112,113,114]. Most likely, the flux of *myo*-inositol used to feed the lipid dependent pathway leading to the production of Ins(1,4,5)P_3_ or the regulation of the salvage pathway used for *myo*-inositol catabolism might be affected in these *mrp* mutants (Figure 1).

Misregulation (in most cases downregulation) of multiple genes for the synthesis and transport/accumulation of phytic acid has also been reported in a number of *lpa* mutants. Decreased expression of *MIPS* and *IMP* has been shown in *lpa2-1*, *lpa3-3* and *M955* barley mutants [107]. A point mutation in the bean phytic acid transporter (*Pvmrp1*) causes a general transcriptional down regulation of the genes of phytic acid pathway, indicating that the *myo*-inositol cellular pool and phytic acid biosynthesis are controlled by phytic acid itself and/or other constituents of the pathway [76]. Similar results have been reported in a more extensive study on the *Arabidopsis* *lpa* mutants *atmik, atitpk1, atitpk4, atipk1, atipk2*β*, atmrp5* and *at2pgk* [35]. In these knock-out mutants, the expression of several genes of the InsP_6_ pathway was affected, confirming that other constituents of InsP_6_ metabolism also modulate the transcription of genes in the InsP_6_ pathway. Another interesting outcome of this study was the observation that the lipid dependent pathway, compared to the lipid independent one, has a more relevant regulatory role in mediating stress response. In fact, defects affecting inositol phosphate kinases (*atipk1*, *atipk2*β, *atitpk1* and *atitpk4-1*) are more relevant for abiotic stress (NaCl, mannitol and H_2_O_2_) sensitivity, than mutations in genes of the early pathway (*atmik1* and *at2pgk*) or in the phytic acid transporter (*atmrp5*).

The pathway for phytic acid biosynthesis is more than a simple, linear addition of phosphate esters, and its complexity is well illustrated by the multiple activities of the inositol tris/tetra kisphosphate kinases (ITPKs). These enzymes have been shown to have multiple substrate specificity, and, in some cases, may also act as isomerases and/or phosphatases of several inositol phosphates [34,39,59]. ITPKs may differ in their tissue and time of expression, as well as their substrate specificity and affinity [32,34,36,37,39,59]. For example, rice and barley ITPKs (OsIpk and HvIpk) show the highest affinity for the Ins(3,4,5,6)P_4_ → Ins(1,3,4,5,6)P_5_ reaction, in agreement with the results obtained for the maize ITPK gene (ZmItpk) [62]. However, the OsItpk and HvItpk proteins also have high activity towards Ins(3,4,5)P_3_ and are able to produce Ins(1,3,4,5,6)P_5_ using an InsP_4_ as intermediate, but, contrary to ZmItpk, they are not capable of phosphorylating Ins(1,3,4,5)P_4_ [34].

## 4. Enzymes and Metabolites of the Phytic Acid Pathway Have Regulatory Roles in Cell Signaling and Plant Processes

Despite its importance, the biological role of InsP_6_ in plants is still poorly understood in both normal and extreme environmental conditions. *Myo*-inositol synthesis and catabolism impact metabolites involved in many critical plant biochemical pathways, such as (i) the production of compatible solutes, like galactinol, raffinose family oligosaccharides, pinitol and cell wall polysaccharides; (ii) the generation of inositol polyphosphates (InsPs), phytic acid and inositol polyphosphate pyrophosphates (PP-InsPs); and (iii) the synthesis of phosphoinositides and the production of Ins(1,4,5)P_3_ (Figure 2). Furthermore, d-glucuronic acid, the primary breakdown product of Ins, is utilized in the synthesis of various cell wall pectic and noncellulosic compounds and ascorbic acid [115,116,117]. A growing body of data is elucidating the roles played by inositol metabolism in diverse plant developmental and physiological processes including signal transduction [113,118], sugar signaling [115], storage and polar transport of auxin [44,119], membrane trafficking [120], abiotic and biotic stress response [48,121], phosphorus homeostasis [55,122], photomorphogenesis [64], chromatin modification and remodeling [122,123], and mRNA nuclear export [124]. Highly phosphorylated inositols (InsP_5_, InsP_6_, InsP_7_, InsP_8_) have also been shown to serve as ligands of plant hormone receptors [125,126,127]. Moreover, several genes, enzymes and compounds for inositol phosphates and, eventually, phytic acid synthesis are part of cytosolic and nuclear metabolic pools with a central role in cellular metabolism. Thus, any perturbations of the pathway, such as those occurring in *lpa* mutants, may significantly impact seed and plant development (Figure 2).

### 4.1. Signal Transduction

Inositol metabolism plays a significant role in a wide range of plant developmental and physiological processes, such as response to diverse stimuli (light, gravitropism, abiotic and biotic stresses), downstream responses to ABA and sugars, and auxin mediated processes, among others, as described below. Key molecules are the well known Ins(1,4,5)P_3_ (InsP_3_) and InsP_6._ Very recently, inositol pyrophosphates (InsP_7_ and InsP_8_) have been proposed as unique signaling molecules involved in energy sensing and metabolism [127,128,129,130,131]. These high energy molecules, containing a linear chain of two (PP) or three (PPP) phosphates linked to an InsP_6_ molecule (Figure 1), have been implicated in determination of the phenotypes of *lpa* mutants, based on their increased expression in seeds of *Arabidopsis* and maize *mrp* mutants (*mrp5* and *lpa1-1* respectively) [129].

In eukaryotes, the *myo*-inositol signaling pathway relies on InsP_3_ as second messenger to trigger intracellular Ca^2+^ release from intracellular stores. When exposed to an external stimulus, the cell responds increasing InsP_3_, which is generated by the action of phospholipase C (PLC). The signal induced by InsP_3_ can be terminated through a catabolic pathway (the so-called “salvage pathway”), in which *myo*-inositol polyphosphate phosphatases (Ptases) remove the 5-phosphate to regenerate *myo*-inositol, or by InsP_3_ removal through sequential phosphorylation mediated by IPK2 (Figure 1). Thus, InsPs and PtdInsPs are interdependent compounds, as PtdInsPs are used as substrates by PLCs to produce InsPs, and InsPs breakdown produces *myo*-inositol used as substrate to produce PtdInsPs. The direct role of InsP_3_ in signaling is in question, as an emerging idea is that InsP_3_ plays a role simply as a precursor of InsP_6_ [121]. In fact, in guard cells, InsP_6_ has been shown to trigger intracellular Ca^2+^ release after ABA addition with an efficiency ≈100 times higher than that of InsP_3_ [118]. Moreover, there is no evidence of a canonical InsP_3_ receptor in plants, although this holds true also for InsP_6_. One possibility is that plants do not possess an InsP-regulated calcium channel, and an entirely different and uncharacterized mechanism allows both InsP_3_ and InsP_6_ to regulate Ca^2+^ release, either simultaneously, or independently [111]. Most likely, both InsP_3_ and InsP_6_ have parallel and overlapping functions in plants, and there is a large body of evidence showing that InsPs impact cellular Ca^2+^ levels. Undoubtedly a strong correlation exists between these two molecules as demonstrated by the finding that transgenic plants and plant cells that constitutively break down InsP_3_ or synthesize PtdInsP_2_ contain changes in InsP_6_: so changes in InsP_3_ are mirrored by changes in InsP_6_ [132,133,134].

Rapid increases in InsP_3_ have been reported in response to myriad stimuli, such as gravitropism, light, salt stress, gibberellic acid, anoxia, cold, heat, drought, and exposure to plant pathogens and elicitors [111,113,114]. Thus, directly or indirectly, InsP_3_ is crucial to signal transduction. Intriguingly, conditions that lower InsP_3_ mediated signals, while revealing predictable phenotypes based on signal transduction paradigms, also showed unexpected phenotypes. For example, *Arabidopsis* plants overexpressing human type I inositol polyphosphate 5-phosphatase (InsP 5Ptase), have a 2%–5% reduction of InsP_3_ and even under stimulation these levels do not show any increase [135]. In normal conditions no obvious phenotypic alterations were detected, while, as expected, InsP_3_-mediated responses, such as gravitropism, were delayed in the InsP 5Ptase overexpressing transgenic plants. Surprisingly, human InsP 5Ptase plants were more drought tolerant [132], an unexpected finding based on the classical InsP_3_ signaling paradigm that predicts decreased levels of InsP_3_ and calcium would decrease stomatal closure and therefore drought tolerance. This finding might explain the contrasting response to ABA during seed germination observed in common bean and *Arabidopsis* *lpa* mutants (see above).

As already mentioned, elevated InsP_3_ has been shown to correlate with downstream responses to ABA and sugars [107,108]. A plant inositol polyphosphate 5-phosphatase (5Ptase13; At1g05630) has been shown to be involved in linking *myo*-inositol signaling to sugar sensing and stress response [136]. The 5Ptase13 protein contains a WD40 repeat region that specifically interacts with a Sucrose non fermenting-1-1Related Kinase (SnRK1.1), which functions as a sensor of energy and stress in plants [137]. When sugars are in limited supply, 5Ptase13 interacts with SnRK1.1 and prevents its proteosomal degradation, resulting in transcriptional induction of genes involved in low nutrients/stress response [110,115]. Several other developmental/signaling defects, altering blue light response, gravitropism, and vesicular trafficking for PIN mediated auxin transport have been reported in *5Ptase13* mutants, indicating that hydrolysis of Ins(1,4,5)P_3_ or PtdIns(4,5)P_2_ are critical for plant development and signaling [138,139].

Inositol polyphosphate kinase (IPK2) is a key component for InsP_3_ turnover, as it phosphorylates Ins(1,4,5)P_3_ successively at the 6- and 3-positions to generate Ins(1,4,5,6)P_4_ and Ins(1,3,4,5,6)P_5_, respectively, and IPK2 has very important regulatory roles, indicated by subcellular localization in both the nucleus and cytoplasm (discussed below). Downregulation of *AtIPK2*α through antisense inhibition has been shown to result in enhanced root growth and pollen germination [56]. Since application of InsP_3_ can enhance root growth, most likely *AtIPK2*α silencing may cause the accumulation of InsP_3_ in addition to the expected depletion of InsP_4_ and InsP_5_.

### 4.2. Vesicle Trafficking and Polar Auxin Transport

A number of *lpa* mutants displays severe defects in embryo and/or plant development, and this often occurs when *MIPS* genes are mutated or their expression is greatly altered [44,45,88] (Table S1). In developing seeds *MIPS* genes are highly expressed, suggesting an important role of *myo*-inositol in seed/embryo development. Using double and triple *mips* mutants of *Arabidopsis*, which display phenotypes resembling those of auxin mutants, it has been shown that MIPS-mediated *de novo* synthesis of *myo*-inositol is essential for maintaining the normal function of endomembrane trafficking and for maintaining endomembrane structure. This is critical for correct auxin transport and thus for correct auxin localization during embryo pattern formation [44]. PtdIns contributes 21% of the phospholipids in nonphotosynthetic plant membranes, and the various phosphorylated forms of PtdIns have critical roles in cytoskeletal rearrangements, membrane trafficking, and organelle labeling. Indeed, the finding that *Arabidopsis* *mips1*/*mips3* double mutants can be rescued by over-expressing the phosphatidyl synthase 2 gene (*AtPIS2*), controlling the entry point for PtdIns synthesis, supports the hypothesis that PtdIns and phosphatidylinositides are essential for endomembrane structure and trafficking [44]. Another study showed that the *atmips1* mutant has reduced levels of PtdIns and altered trafficking of the auxin efflux carrier PIN2 [98,106], suggesting that lower PtdIns content negatively impacts on vesicular trafficking of PIN2. Interestingly, the *atmips1* phenotype was evident only if plants were grown under high light intensity conditions (higher than160 μmol m^−2^·s^−1^) [44]. Although currently not intensely studied, it is likely that not only PIN proteins but also many other membrane proteins will depend on PtdIns to reach their membrane destinations. This may indicate other obvious and broad ramifications of inositol metabolisms in plant function [100]. The critical role of *myo*-inositol as precursor of PtdIns species is also confirmed by the finding that InsP_3_ is linked to Ca^2+^ signaling and control of directional auxin transport mediated by polar PIN auxin transporters [140]. During a screen for *suppressors of* *PIN1 overexpression*, Zhang *et al.*, isolated an *Arabidopsis* mutant (*supo1*) affected in an inositol phosphate 1-phosphatase known as SAL1/FRY1 [114]. The mutant had increased InsP_3_ and cytosolic Ca^2+^ levels and was able to restore a wild type phenotype in *35S::PIN1* seedlings. PIN overexpressing plants have short roots, agravitropic root growth, and elevated auxin in root tips, presumably caused by the ectopic presence of basally localized PIN1 in the epidermal cells.

Involvement of InsP metabolism in auxin mediated processes is also provided by the finding that plants over-expressing the *AtIPK2*β gene show an attenuation of the inhibition of IAA induced primary root elongation. This indicates that AtIPK2β can negatively regulate auxin signaling [57]. At least in part, this regulation is transcriptional, since *AtIPK2*β over-expressing plants repress the expression of *CYP83B1* (involved in auxin biosynthesis), *MAX4* and *SPS* (required for auxin-mediated bud inhibition and outgrowth), and stimulate *PIN4* expression [57].

### 4.3. Biotic and Abiotic Stress Response

Limiting PtdIns synthesis and/or InsP_6_ content may also impact plant defense response. The *Arabidopsis* *atmips1* mutant showed enhanced resistance to pathogens and spontaneous cell death, thus implying that MIPS is a repressor of programmed cell death (PCD) [97]. In this mutant decreased contents of *myo*-inositol, ascorbic acid and PtdIns correlated with elevated ceramide levels, sphingolipid precursors associated to cell death. The hypothesis is that spontaneous cell death is the result of altered oxidative stress sensitivity, induced by changes in *myo*-inositol, galactinol, and ascorbic acid, along with elevated ceramides and hydroxyceramides that result from decreased PtdIns availability for sphingolipid production [111]. *Myo*-inositol metabolism and defense response are also linked to carbohydrate metabolism, as a mutation in the hexokinase 1 (HXK1) enzyme has been shown to suppress *mips1* light-dependent PCD, indicating an epistatic relationship between *mips1* and *hxk1* mutants [141].

Like other enzymes of the InsP_6_ biosynthetic pathway (see below), MIPS has a double cellular localization: cytosolic and nuclear. Interestingly, in a recent work in *Arabidopsis*, it has been demonstrated that MIPS1 protein binds directly to its own promoter to stimulate transcription by locally inhibiting the activation of ARABIDOPSIS TRITHORAX-RELATED 5 and 6 (ATXR5 and 6)-dependent heterochromatin marks generated by a transposable element [123]. Upon activation of pathogen response, elicited by bacterial flagellin (flg22) treatment, the inhibitory action of MIPS1 on ATXR5/6 is alleviated and expression of *MIPS1* decreases. This dual function of MIPS1 may ensure *MIPS1* gene expression under normal growth conditions, and its down-regulation during pathogen attack to induce PCD [123]. It would be very interesting to verify if such regulatory mechanism is specific to *Arabidopsis* or is present in other plants.

Impaired resistance to wounding and herbivory has also been reported in transgenic plants in which inositol polyphosphates are globally reduced through expressing a human type I InsP 5-Ptase. In this case, plants treated with flg22 show impaired Ca^2+^ elevation, accompanied by a decrease in the expression of several defense related genes, suggesting that the inability to propagate an InsP_3_ signal is the primary basis for the altered defense response observed [142]. Other evidence for the involvement of InsPs in wound signaling comes from work on *atipk1-1* mutants, which exhibit a pathogen hypersensitive phenotype and increased defense capability via jasmonate receptor COI1-mediated processes, including wound-induced gene expression, defense against caterpillars or root growth inhibition by jasmonate [48,143].

Perturbations of the InsP_6_ pathway have been reported to alter plant response to environmental stimuli. For instance, heterologous expression of *AtIPK2*β in tobacco leads to improved tolerance to diverse abiotic stresses (osmotic, drought, freezing temperature, oxidative stress) [58]. Similar results have been obtained by over-expressing the *IbMIPS1* gene in sweet potato (*Ipomoea batatas* L. Lam.). Transgenic plants showed significantly enhanced salt and drought tolerance, and stem nematode resistance. Following exposure to salt and nematode stresses, transcriptome analysis revealed up-regulation of *MIPS* and *IMP* genes, together with genes for phosphatidylinositol and ABA signaling pathways, stress responses and ROS-scavenging [144].

### 4.4. Nuclear Functions and Regulation of Phosphorus Homeostasis

A central issue in InsP metabolism is the cellular localization of InsP pools. Dual localization, cytosolic and nuclear, of inositol phosphates and of their biosynthetic enzymes is well documented in yeast [145] and has been demonstrated in plants for many enzymes of the InsP_6_ pathway, including a number of phosphatidylinositol kinases, AtMIPS1, AtIPK2α, AtIPK2β, AtIPK1 and AtITPK1 [56,64,98,122,123,146].

In yeast, the transcriptional activation of *PHO* genes in response to P_i_ deficiency is coordinated with regulated chromatin remodeling [145]. Furthermore, a number of InsPs species have been shown to participate to transcriptional gene regulation via chromatin remodeling and histone modification [147]. For example, proper expression of *INO1*, coding inositol 1-phosphate synthase, involves integration of INO80 and SNF, and ISW2, which act as positive or negative regulators of transcription, respectively. InsP_4_ and InsP_5_ have also been shown to stimulate nucleosome mobilization by the SWI/SNF complex. On the contrary, InsP_6_ inhibits nucleosome mobilization by NURF, ISW2 and INO80 complexes [148]. The yeast IPK2 and its products, InsP_4_ and InsP_5_, are involved in transcriptional regulation in response to environmental and nutritional stresses. IPK2 has been shown to be allelic to Arg82/ArgRIII, a component of the ArgR-Mcm1 transcriptional complex that regulates gene expression for arginine metabolism [149]. IPK2 is also required for the induction of some phosphate responsive genes (*PSR*), like *PHO5*, by modulating the chromatin remodeling complexes SWI/SNF and INO80, under normal P_i_ supply [150]. Xia and coworkers [146] demonstrated that *Arabidopsis* *AtIPK2*β is able to complement a yeast *Arg82/ArgRIII* mutant lacking a functional ArgR-Mcm1 transcription complex. However, no data are available to support a similar role for AtIPK2β in *PSR* gene induction, as was shown in yeast.

Plant P_i_ homeostasis is a highly regulated process, and involves the perception of P_i_ present in the environment, followed by acquisition, remobilization and recycling of P_i_ [151]. The *Arabidopsis* mutant *atipk1-1* exhibits an 83% reduction in seed InsP_6,_ along with an increase in InsP_4_ and InsP_5_ in seed and vegetative tissues. Furthermore, *atipk1-1* plants show longer root hairs and aberrant phosphate sensing. The plants behave as if they were in a phosphorous-limiting environment, indicating a role of IPK1 in the regulation of P_i_ acquisition machinery [55].

In a recent work, transcriptional analysis of roots of *atipk1-1* mutants showed perturbations of a subset of P_i_ starvation responsive genes, together with increased expression of genes involved in P_i_ uptake, allocation and remobilization [122]. The authors observed that the transcriptional activation correlate with reduction of the chromatin association of histone variant H2A.Z. In yeast the P_i_ responsive genes *PHO5* and *PHO84* require InsP_4_ and InsP_5_ for proper remodeling of chromatin structure [150], thus it might be speculated that in plants, specific InsPs species serve as signals and regulate the eviction of H2A.Z from *PSR* genes.

Perturbation of P_i_ and sulfate (SO_4_) homeostasis and signaling have been observed in *lpa* plants obtained by over-expressing a bacterial phytase in *Arabidopsis* [152]. These plants (*PHY-US417*) showed up to 50% and 45% increases in shoot P_i_ and SO_4_ concentrations, respectively, and improved plant growth with enhanced root growth capacity in P_i_ deficiency. These findings were supported by expression analysis of a subset of genes coding for P_i_ transporters (*PHT1,1*, *PHT1,4*, *PHO1* and *PHO1,H1*) and SO_4_ (*SULTR1,2*), the expression of which was upregulated in both overexpressing *PHY-US417* and *atipk1-1* plants. Interestingly, overexpressing *PHY-US417* transgenic plants and the *atipk1-1* also showed a stronger remobilization of iron during germination.

One of the key points for proper gene expression is the regulation of nuclear export of mRNA, a process that requires the directional translocation of mRNA-ribonucleoprotein particles (mRNPs) through nuclear pore complexes (NPCs). In eukaryotes, Gle1 is a component of the NPC. In yeast, Gle1 and its cofactor InsP_6_ activate the DEAD-box ATPase, Dbp5, to allow mRNA export at the NPC. Gle1 is also found in the cytosol, where it plays a role in translation initiation and termination in Dbp5-independent and -dependent manners, respectively [145]. In a very recent work, Lee *et al.* [124] showed that plant Gle1, in conjunction with InsP_6_, functions as an activator of the ATPase/RNA helicase LOS4 (low expression of osmotically responsive genes 4, homolog of yeast Dbp5), which is involved in mRNA export in plants, supporting the Gle1-InsP_6_-Dbp5 paradigm proposed in yeast. Interestingly, an *ipk1* mutant has been shown to be defective in nuclear mRNA export, however, the ectopic expression of Gle1 variants with enhanced InsP_6_ sensitivity was able to rescue the mRNA export defect of the *ipk1* mutant. Moreover, a significant improvement of vegetative growth, seed yield, and seed performance of the mutant was observed, suggesting that Gle1 is an important factor responsible for mediating InsP_6_ functions in plant growth and reproduction [124].

### 4.5. Hormonal Signaling

In recent years, it was discovered that two important phytohormone receptors, TIR1 and COI1, required for auxin and jasmonic acid (JA) signaling, respectively, contain InsPs as structural cofactors, thus widening the regulatory roles involving the InsP_6_ pathway [126,153]. TIR1 is part of the ubiquitin E3 ligase complex SCF^TIR1^. Upon auxin binding, it recruits specific transcriptional repressors (the Aux/IAA repressors) for ubiquitination by the SCF complex. This marking process leads to the degradation of the repressors by the proteasome, alleviating repression and leading to expression of specific auxin responsive genes [154]. The determination of the crystal structure of TIR1 revealed the presence of an InsP_6_ molecule bound in close proximity to the auxin binding pocket [126]. In a similar way, COI1 is the F-box component of a SCF ubiquitin E3 ligase complex that recruits Jasmonate ZIM-domain (JAZ) transcriptional repressors upon binding to the bioactive JA-isoleucin conjugate (JA-Ile). This triggers JAZ polyubiquitination and subsequent proteasomal degradation, and results in de-repression of MYC2 dependent transcription of jasmonate repsponsive genes [154]. Analysis of the crystal structure of the JA receptor revealed a binding pocket comprised of COI1, bound to JAZ, and containing an InsP_5_ molecule as a structural cofactor [154]. *Arabidopsis* *ipk1* mutants, that have elevated levels of InsP_5_ and display enhanced wound-induction of various defense genes, were found to be more sensitive towards exogenous methyl-JA, and exhibited increased defensive capacity against caterpillar herbivory [143], supporting the importance of InsP_5_ contribution to COI1 function. However, very recently, a link between InsP_8_ and jasmonate-dependent defense has been discovered. The authors do not rule out that other inositol polyphosphates, other than InsP_8_, may influence assembly of the jasmonate receptor complex [127], but insect larvae feeding on *Arabidopsis* *vih2* mutant plants (unable to synthesize InsP_8_, Figure 1) showed a significant weight increase compared with larvae feeding on control plants, indicating that VIH2 plays a role in activating defenses that interfere with insect herbivore development. Furthermore, molecular data indicate that *vih2* plants are defective in jasmonate perception.

## 5. Conclusions 

This object of this review is to integrate current knowledge about different aspects of phytic acid pathway and *lpa* mutants with the most recent literature concerning the regulatory roles of the multiple components of the pathway in cell signaling and plant processes. In the last decade, the range of important crop plants with *lpa* mutants has expanded rapidly. Known *lpa* mutants have seed phytic acid reductions ranging from 10% to 90%, and in many cases their improved value in animal and human nutrition has been demonstrated. However, good agronomic performance and yield stability are still challenging for *lpa* mutants in many crops. In no case were these mutants due to a spontaneous mutation, underlying the important role of InsP_6_ pathway for the plant. The increasing number of plant processes in which phytic acid and its metabolism have been shown to play a key role clearly indicates that we need to increase our knowledge of the role of InsPs and phytic acid in the integration and functioning of metabolic and hormonal signaling pathways and in response to biotic and abiotic stresses. This knowledge will be fundamental to understand how far we may go to obtain stable and productive *lpa* mutants in different crops and how to drive their genetic improvement.

## Supplementary Materials

Supplementary materials can be accessed at: http://www.mdpi.com/2223-7747/4/4/728/s1.

## Abbreviations

Ins, *myo*-inositol; InsPs, inositol phosphates; InsP_6_*, myo*-inositol-1,2,3,4,5,6-hexa*kis*phosphate; MIPS, *myo*-inositol-3-phosphate synthase; IMP, *myo*-inositol-phosphate monophosphatase; MIK, *myo*-inositol kinase; IPK1, inositol polyphosphate 2-kinase; ITPK, the inositol 1,3,4-trisphosphate 5-/6-kinase; IPK2, inositol 1,4,5-tris-phosphate kinase.
