# Supplementary material for: Seed Biofortification and Phytic Acid Reduction: A Conflict of Interest for the Plant?
Source: Plants (Basel). 2015 Nov 20;4(4):728–55. doi: 10.3390/plants4040728 (PMC4844270; doi:10.3390/plants4040728)
Supplement: Supplementary file 1 [file plants-04-00728-s001.pdf]

Supplementary Materials

Table S1. Summary of the *lpa* mutants isolated in different species. nd, not determined; OE, overexpression; RNAi, RNA interference.

Gene Function	Species	Locus	Origin of Mutation	Mutant (Ecotype)	Seed PA Reduction	Metabolites and/or Genes DOWN Regulated	Metabolites and/or Genes UP Regulated	Other Phenotypic Alterations	Ref.
MIPS1	*Arabidopsis thaliana*	At4g39800	T-DNA insertion	*atips1-1* (Col), *atips1-2* (Ws)	nd	phytic acid, *myo*-inositol and galactinol in leaves		reduced cell proliferation in seedling causing strong modification of the root cap and shorter seedling; abnormal cotyledons; leaf lesions on adult plants modulated by light intensity and developmental stages, due to SA-dependent PCD (increase in SA content); increased resistance to oomycete	[1,2]
MIPS1	*Arabidopsis thaliana*	At4g39800	T-DNA insertion	*mips1-2* (Col), *mips1-4* (C-24), *mips1-5* (Col)	nd			defects in: embryogenesis, cotyledon vein patterning, epidermal cell division, root growth and gravitropism, root cap cell patterning, apical dominance, auxin response	[3]
MIPS1	*Arabidopsis thaliana*	At4g39800	T-DNA insertion	*mips1-2* (Col), *mips1-3* (Col)	nd	*myo*-inositol, ascorbic acid, phosphatidyl inositols	ceramides	smaller plants, curly leaves, spontaneus lesions, increased sensitivity to ABA and NaCl in germination	[4]
MIPS2	*Arabidopsis thaliana*	At2g22240	T-DNA insertion	*atips2* (Col)	none	phytic acid in leaves		increased susceptibility to viruses, fungi, bacteria	[1,5,6]
MIPS3	*Arabidopsis thaliana*	At5g10170	T-DNA insertion	*atips3* (Col)	none			lethal	[2,5,6]
MIPS1	*Glycine max*	Glyma18g02210	γ-rays	*Gm-lpa-TW75-1*	50%	*myo*-inositol, galactinol, raffinose, stachyose, galactopinitol A, galactopinitol B, fagopyritol B1, galactinol	sucrose, glycerol, sorbitol	reduced field emergence (seeds produced in subtropical environment)	[7–9]
MIPS1	*Glycine max*	Glyma18g02210	*N*-nitroso-*N*-methylurea	*LR33*	50%	*myo*-inositol, raffinose, stachyose	sucrose	reduced field emergence (seeds produced in subtropical environment)	[10,11]

Table S1. *Cont.*

Gene Function	Species	Locus	Origin of Mutation	Mutant (Ecotype)	Seed PA Reduction	Metabolites and/or Genes DOWN Regulated	Metabolites and/or Genes UP Regulated	Other Phenotypic Alterations	Ref.
MIPS1	*Glycine max*	Glyma18g02211		*V99-5089*	50%		sucrose		[12,13]
MIPS1	*Glycine max*	Glyma18g02212	RNAi	Δ*GmMIPS*	35%–95%			seed abortion in fully silenced seeds	[14]
MIPS	*Solanum tuberosum*		antisense	antisense *StIPS*	nd	Phytic acid in tubers, *myo*-inositol (leaf and tuber), galactinol, raffinose (leaf), sucrose, starch (tuber)	sucrose, starch (leaf)	reduced apical dominance, delayed flowering, increased leaf thickness, precocious leaf senescence, mini tubers, no anthocyanins in tuber skin, increased susceptibility to virus	[1,15]
MIPS	*Oryza sativa*	Os03g09250	antisense	antisense *RINO1* under olesin 18kDa promoter (*Ole18*)	75%			affected only embryo and aleurone layer; no negative effects on seed weight, germination and plant growth	[16]
IMP	*Arabidopsis thaliana*	At3g02870	T-DNA insertion	*vtc4*	nd	*myo*-inositol, ascorbic acid	galactose		[17]
IMT from *M. crystallinum*	*Brassica napus*		over-expression	pN*IMT* (*IMT* under napin promoter) and pPh*IMT* (*IMT* under phaseolin promoter)	20%–30%	galactinol, raffinose	galactose, stachyose, sucrose		[18]
MIK	*Arabidopsis thaliana*	At5g58730	T-DNA insertion	*atmik-1*	62%	IPK2β, ITPK1, ITPK4, MRP, 2PGK		reduced germination on NaCl, mannitol, H_2O_2	[5]
MIK	*Arabidopsis thaliana*	At5g58730	T-DNA insertion	*atmik-2*	66%				[5]
MIK	*Zea mays*	GRMZM2G361593	*Mu* insertion	*lpa3*	45%	lower InsPs	*myo*-inositol		[19]
MIK + ITPK	*Zea mays*	GRMZM2G361593 GRMZM2G456626	*Mu* insertion	*lpa2-lpa3*	66%		molar increase of lower InsPs		[19]
MIK	*Oryza sativa*	Os03g52760	γ-rays + sodium azide	*Os-lpa-XS110-1*	64%	InsP	*myo*-inositol, raffinose, galactose, galactinol, fructose, glucose	seed dry weight reduction, low grain yield, reduced germination (particularly after aging), reduced field emergence. Proteomic study shows 23 proteins UP-regulated in the mutant	[20–23]
MIK	*Oryza sativa*		EMS	*lpa N15-186*	75%		*myo*-inositol		[24]

Table S1. *Cont.*

Gene Function	Species	Locus	Origin of Mutation	Mutant (Ecotype)	Seed PA Reduction	Metabolites and/or Genes DOWN Regulated	Metabolites and/or Genes UP Regulated	Other Phenotypic Alterations	Ref.
2-PGK	*Arabidopsis thaliana*	At5g60760	T-DNA insertion	*Oslpa1 like*	66%	MIK, ITPK1, ITPK4, MRP	IPK2β, IPK1	reduced germination on NaCl, mannitol, H_2O_2	[25,5]
2-PGK	*Oryza sativa*	Os02g57400	γ-rays	*XQZ-1*	40%		molar increase of lower InsPs	seed dry weight reduction, low grain yield, reduced germination (particularly after aging)	[22]
2-PGK	*Oryza sativa*	Os02g57400	γ-rays	*KBNT lpa1-1*	40%		molar increase of lower InsPs	normal plants, reduced size of globoids	[24]
IPK2β	*Arabidopsis thaliana*	At5g61760	T-DNA insertion	*ipk2β-1*	35%				[26]
IPK2β	*Arabidopsis thaliana*	At5g61760		*ipk2β-2*	48%	MIK, ITPK1, ITPK4, 2PGK	IPK1, MRP	reduced germination on NaCl, mannitol, H_2O_2	[5]
ITPK1	*Arabidopsis thaliana*	At5g16760	T-DNA insertion	*atitpk1*	46%	MIK, 2PGK	IPK1, MRP	reduced germination on NaCl, mannitol, H_2O_2	[5]
ITPK4	*Arabidopsis thaliana*	At2g43980	T-DNA insertion	*atitpk4-1*	51%	MIK, 2PGK	IPK1, MRP	reduced germination on NaCl, mannitol, H_2O_2	[5]
ITPK4	*Arabidopsis thaliana*	At2g43980	T-DNA insertion	*atitpk4-2*	40%				[5]
ITPK	*Zea mays*	GRMZM2G456626	*Mu* insertion	*lpa2*	30%		molar increase of lower InsPs	seed dry weight reduction	[27,28]
ITPK	*Oryza sativa*	Os09g34300	EMS	*itpk*	46%–68%			seed dry weight reduction, low grain yield, reduced germination	[29]
IPK1	*Arabidopsis thaliana*	At5g42810	T-DNA insertion	*ipk1-1*	83%			altered Pi homeostasis (smaller leaves and epinasty at standard Pi concentration, longer roots at lower Pi concentration, attenuated ability to sense Pi increase)	[26]
IPK1	*Arabidopsis thaliana*	At5g42810	T-DNA insertion	*ipk1-1*	49%	MIK	IPK2β, ITPK1, ITPK4, MRP, 2PGK	reduced germination on NaCl, mannitol, H_2O_2	[5]
IPK1	*Arabidopsis thaliana*	At5g42810	T-DNA insertion	*ipk1-1*	nd	phytic acid in leaves		increased susceptibility to viruses, fungi, bacteria	[1]

Table S1. *Cont.*

Gene Function	Species	Locus	Origin of Mutation	Mutant (Ecotype)	Seed PA Reduction	Metabolites and/or Genes DOWN Regulated	Metabolites and/or Genes UP Regulated	Other Phenotypic Alterations	Ref.
IPK1	*Arabidopsis thaliana*	At5g42810	T-DNA insertion	*ipk1-1*	nd			increased Pi uptake and increased root to shoot translocation, Pi deficiency-like root system architecture	[30]
IPK1	*Arabidopsis thaliana*	At5g42810	T-DNA insertion	*ipk1-1*	nd			early flowering, seed yield 52% of wt, reduced sensitivity to salt stress	[31]
IPK1	*Arabidopsis thaliana*	At5g42810	T-DNA insertion	*ipk1-2*	nd			not viable	[30]
IPK1	*Glycine max*	Glyma14g07880	γ-rays	*Gm-lpa-ZC-2*	46%		*myo*-inositol, syringic acid, total isoflavones, molar increase of lower InsPs	shorter plants, but no yield reduction	[7]
IPK1	*Zea mays*	GRMZM2G067299	ZFN-DNA editing	*ipk1*	50%				[32]
IPK1	*Oryza sativa*	Os04g56580	RNAi under Oleosin 18 promoter	*ipk1*	70%			unchanged levels of *myo*-inositol	[33]
MRP	*Arabidopsis thaliana*	At1g04120	T-DNA insertion	*atmrp5-1* (Ws)	nd			on 0.5 × MS decreased root growth and increased lateral root formation, on 1 × MS reverse phenotype, increased level of auxin in root, no effect of sulfonylurea glibenclamide on stomatal opening	[34]

Table S1. *Cont.*

Gene Function	Species	Locus	Origin of Mutation	Mutant (Ecotype)	Seed PA Reduction	Metabolites and/or Genes DOWN Regulated	Metabolites and/or Genes UP Regulated	Other Phenotypic Alterations	Ref.
MRP	*Arabidopsis thaliana*	At1g04120	T-DNA insertion	*atmrp5-1* (Ws)	nd			more resistant to drought stress, increased water use efficiency, stomata closer under light, no sensitivity to Ca2+ and ABA on stomatal closure, no sensitivity to auxin for opening under darkness, reduced ABA sensitivity during germination	[35]
MRP	*Arabidopsis thaliana*	At1g04120	T-DNA insertion	*atmrp5-1* (Ws)	nd			impairment in activation of slow S-type anion channels in the plasma membrane of GCs	[36]
MRP	*Arabidopsis thaliana*	At1g04120	T-DNA insertion	*atmrp5-2* (Ws)	nd			hypersensitivity to salt stress	[37]
MRP	*Arabidopsis thaliana*	At1g04120	T-DNA insertion	*mrp5-1* (Ws), *mrp5-2* (Col)	73%–80%	34%–37% reduction in total P; reduction in Na, Mg, Ca, K content			[38]
MRP	*Arabidopsis thaliana*	At1g04120	T-DNA insertion	*atmrp5-1 and 5-2* (Col)	73%–80%	MIK, IPK2βITPK1, ITPK4, 2PGK	IPK1	reduced germination on NaCl, mannitol, H_2O_2	[5]
MRP	*Arabidopsis thaliana*	At1g04120	T-DNA insertion	*atmrp5* (Col)	nd			enhanced lateral roots and root hair growth	[30]
MRP	*Arabidopsis thaliana*	At1g04120	T-DNA insertion	*atmrp5*	nd			increase in InsP5, InsP7 and InsP8 in siliques	[39]
MRP	*Glycine max*	Glyma03g32500 Glyma19g35230	EMS	*CX1834*	80%	palmitate, stearate, oil		reduced seedling and field emergence (seeds produced in subtropical environment)	[12,13, 40–43]
MRP	*Phaseolus vulgaris*	Phvul001g165500	EMS	*lpa1*	90%	*myo*-inositol, raffinosaccharides, MIPS, IMP, IPK2,ITPKα, IPK1		down-regulation of genes (MIPS, IMP, IPK2, ITPKα, IPK1) for PA biosynthetic pathway, germination hypersensitivity to ABA	[44]

Table S1. *Cont.*

Gene Function	Species	Locus	Origin of Mutation	Mutant (Ecotype)	Seed PA Reduction	Metabolites and/or Genes DOWN Regulated	Metabolites and/or Genes UP Regulated	Other Phenotypic Alterations	Ref.
MRP	*Zea mays*	GRMZM5G820122	EMS	*lpa1-241*, *lpa1-7*	90%	*myo*-inositol, MIPS	Mg, K	seed dry weight reduction, down-regulation of MIPS, altered morphology of globoids (number and size), altered embryo development, size, germination, seedling growth rate, ear size, increased susceptibility to oxidative stress	[45–50]
MRP	*Zea mays*	GRMZM5G820123	EMS	*lpa1-1*	66%	MIPS	*myo*-inositol	reduced germination, reduced vigour under stress conditions, altered morphology of globoids (number and size)	[27,32, 51–53]
MRP	*Oryza sativa*	Os03g04920	γ-rays + sodium azide	*Os-lpa-XS110-2*	20%	*myo*-inositol, raffinose, galactose	24-methylencycloartanol (steroid)	seed dry weight reduction, low grain yield, reduced germination (particularly after aging), reduced field emergence.	[20–22, 54]
MRP	*Oryza sativa*	Os03g04920	γ-rays + sodium azide	*Os-lpa-XS110-3*	100%		*myo*-inositol	(lethal)	[20,54]
MRP	*Oryza sativa*	Os03g04920	T-DNA insertion	*4A-02500*	90%		*myo*-inositol	(lethal)	[20,53]
PAP	*Arabidopsis thaliana*	At3g07130	over-expression	*OE-PAP15*	nd	phytic acid in leaves	ascorbic acid	altered salt, osmotic stress, and ABA sensitivities, enhanced salt tolerance, and decreased abscisic acid sensitivity	[55]
Sultr3;3	*Hordeum vulgare*		sodium azide	*lpa1-1 (M422)*	50%	IMP	Increase in free Pi and small increases in Ins(1,2,3,4,6)P_5	reduced test weight and percentage plump kernels, mutation affects only aleurone. Breeding can abolish/reduce negative traits	[56–60]
Sultr3;3	*Oryza sativa*	Os04g0652400	γ-rays	*MH86-1*	44%		molar increase in free Pi	seed dry weight reduction, low grain yield, reduced germination (particularly after aging)	[20,22]

Table S1. *Cont.*

Gene Function	Species	Locus	Origin of Mutation	Mutant (Ecotype)	Seed PA Reduction	Metabolites and/or Genes DOWN Regulated	Metabolites and/or Genes UP Regulated	Other Phenotypic Alterations	Ref.
unknown	*Pisum sativum*	unknown	EMS	*1-150-81*; *1-2347-144*	60%–65%		molar increase in free Pi	decreased seed weight, decreased yield	[61,62]
unknown	*Hordeum vulgare*	unknown	sodium azide	*lpa3-3 (M635)*	65%	IMP, MIPS	*myo*-inositol, galactinol	reduced test weight and percentage plump kernels, reduced yield. Breeding can abolish/reduce negative traits	[56–59]
unknown	*Hordeum vulgare*	unknown	sodium azide	*M955*	90%	IMP, MIPS	*myo*-inositol, raffinose, galactinol, sucrose	reduced test weight and percentage plump kernels, reduced yield. Breeding can abolish/reduce negative traits	[56,57, 59,63]
unknown	*Oryza sativa*	unknown	γ-rays	*Z9B-1*	45%		low increase in free Pi	seed dry weight reduction, low grain yield, reduced germination (particularly after aging)	[20,22]

References

1. Murphy, A.; Otto, B.; Brearley, C.; Carr, J.; Hanke, D. A role for inositol hexakisphosphate in the maintenance of basal resistance to plant pathogens. *Plant J.* **2008**, *56*, 638–652.
2. Meng, P.; Raynaud, C.; Tcherkez, G.; Blanchet, S.; Massoud, K.; Domenichini, S.; Henry, Y.; Soubigou-Taconnat, L.; Lelarge-Trouverie, C.; Saindrenan, P.; *et al*. Crosstalks between *myo*-inositol metabolism, programmed cell death and basal immunity in *Arabidopsis*. *PLoS ONE* **2009**, *4*, doi:10.1371/journal.pone.0007364.
3. Chen, H.; Xiong, L. *Myo*-inositol-1-phosphate synthase is required for polar auxin transport and organ development. *J. Biol. Chem.* **2010**, *285*, 24238–24247.
4. Donahue, J.; Alford, S.; Torabinejad, J.; Kerwin, R.; Nourbakhsh, A.; Ray, W.; Hernick, M.; Huang, X.; Lyons, B.; Hein, P.; *et al*. The *Arabidopsis thaliana myo*-inositol 1-phosphate synthase1 gene is required for *myo*-inositol synthesis and suppression of cell death. *Plant Cell* **2010**, *22*, 888–903.
5. Kim, S.; Tai, T. Identification of genes necessary for wild-type levels of seed phytic acid in *Arabidopsis thaliana* using a reverse genetics approach. *Mol. Genet. Genom.* **2011**, *286*, 119–133.
6. Luo, Y.; Qin, G.; Zhang, J.; Liang, Y.; Song, Y.; Zhao, M.; Tsuge, T.; Aoyama, T.; Liu, J.; Gu, H.; *et al*. D-*myo*-inositol-3-phosphate affects phosphatidylinositol-mediated endomembrane function in *Arabidopsis* and is essential for auxin-regulated embryogenesis. *Plant Cell* **2011**, *23*, 1352–1372.
7. Yuan, F.; Zhao, H.; Ren, X.; Zhu, S.; Fu, X.; Shu, Q. Generation and characterization of two novel low phytate mutations in soybean (G*lycine max* L. Merr.). *Theor. Appl. Genet.* **2007**, *115*, 945–957.
8. Frank, T.; Nörenberg, S.; Engel, K.H. Metabolite profiling of two novel low phytic acid (*lpa*) soybean mutants. *J. Agric. Food Chem.* **2009**, *57*, 6408–6416.
9. Yuan, F.; Zhu, D.; Deng, B.; Fu, X.; Dong, D.; Zhu, S.; Li, B.; Shu, Q. Effects of two low phytic acid mutations on seed quality and nutritional traits in soybean (*Glycine max* L. Merr). *J. Agric. Food Chem.* **2009**, *57*, 3632–3638.
10. Hitz, W.; Carlson, T.; Kerr, P.; Sebastian, S. Biochemical and molecular characterization of a mutation that confers a decreased raffinosaccharide and phytic acid phenotype on soybean seeds. *Plant Physiol.* **2002**, *128*, 650–660.
11. Meis, S.; Fehr, W.; Schnebly, S. Seed source effect on field emergence of soybean lines with reduced phytate and raffinose saccharides. *Crop Sci.* **2003**, *43*, 1336–1339.
12. Maupin, L.; Rosso, M.; Rainey, K. Environmental effects on soybean with modified phosphorus and sugar composition. *Crop Sci.* **2011**, *51*, 642–650.
13. Gao, Y.; Biyashev, R.; Maroof, M.; Glover, N.; Tucker, D.; Buss, G. Validation of low-phytate qtls and evaluation of seedling emergence of low-phytate soybeans. *Crop Sci.* **2008**, *48*, 1355–1364.
14. Nunes, A.; Vianna, G.; Cuneo, F.; Amaya-Farfan, J.; de Capdeville, G.; Rech, E.; Aragao, F. RNAi-mediated silencing of the *myo*-inositol-1-phosphate synthase gene (*GmMIPS1*) in transgenic soybean inhibited seed development and reduced phytate content. *Planta* **2006**, *224*, 125–132.

15. Keller, R.; Brearley, C.; Trethewey, R.; Muller-Rober, B. Reduced inositol content and altered morphology in transgenic potato plants inhibited for 1D-*myo*-inositol 3-phosphate synthase. *Plant J.* **1998**, *16*, 403–410.
16. Kuwano, M.; Mimura, T.; Takaiwa, F.; Yoshida, K. Generation of stable "low phytic acid" transgenic rice through antisense repression of the 1D-*myo*-inositol 3-phosphate synthase gene (*RINO1*) using the 18-kDa oleosin promoter. *Plant Biotechnol. J.* **2009**, *7*, 96–105.
17. Torabinejad, J.; Donahue, J.; Gunesekera, B.; Allen-Daniels, M.; Gillaspy, G. VTC4 is a bifunctional enzyme that affects *myo*-inositol and ascorbate biosynthesis in plants. *Plant Physiol.* **2009**, *150*, 951–961.
18. Dong, J.; Yan, W.; Bock, C.; Nokhrina, K.; Keller, W.; Georges, F. Perturbing the metabolic dynamics of *myo*-inositol in developing brassica napus seeds through *in vivo* methylation impacts its utilization as phytate precursor and affects downstream metabolic pathways. *BMC Plant Biol.* **2013**, *13*, 84, doi:10.1186/1471-2229-13-84.
19. Shi, J.; Wang, H.; Hazebroek, J.; Ertl, D.; Harp, T. The maize *low-phytic acid 3* encodes a *myo*-inositol kinase that plays a role in phytic acid biosynthesis in developing seeds. *Plant J.* **2005**, *42*, 708–719.
20. Liu, Q.; Xu, X.; Ren, X.; Fu, H.; Wu, D.; Shu, Q. Generation and characterization of low phytic acid germplasm in rice (*Oryza sativa* L.). *Theor. Appl. Genet.* **2007**, *114*, 803–814.
21. Frank, T.; Meuleye, B.; Miller, A.; Shu, Q.; Engel, K. Metabolite profiling of two low phytic acid (*lpa*) rice mutants. *J. Agric. Food Chem.* **2007**, *55*, 11011–11019.
22. Zhao, H.; Liu, Q.; Fu, H.; Xu, X.; Wu, D.; Shu, Q. Effect of non-lethal low phytic acid mutations on grain yield and seed viability in rice. *Field Crop. Res.* **2008**, *108*, 206–211.
23. Emami, K.; Morris, N.J.; Cockell, S.J.; Golebiowska, G.; Shu, Q.Y.; Gatehouse, A.M. Changes in protein expression profiles between a low phytic acid rice (*Oryza sativa* L. Ssp. Japonica) line and its parental line: A proteomic and bioinformatic approach. *J. Agric. Food Chem.* **2010**, *58*, 6912–6922.
24. Kim, S.; Andaya, C.; Newman, J.; Goyal, S.; Tai, T. Isolation and characterization of a low phytic acid rice mutant reveals a mutation in the rice orthologue of maize MIK. *Theor. Appl. Genet.* **2008**, *117*, 1291–1301.
25. Kim, S.; Tai, T. Genetic analysis of two *OsLpa1-like* genes in *Arabidopsis* reveals that only one is required for wild-type seed phytic acid levels. *Planta* **2010**, *232*, 1241–1250.
26. Stevenson-Paulik, J.; Bastidas, R.; Chiou, S.; Frye, R.; York, J. Generation of phytate-free seeds in *Arabidopsis* through disruption of inositol polyphosphate kinases. *Proc. Natl. Acad. Sci. USA* **2005**, *102*, 12612–12617.
27. Raboy, V.; Gerbasi, P.; Young, K.; Stoneberg, S.; Pickett, S.; Bauman, A.; Murthy, P.; Sheridan, W.; Ertl, D. Origin and seed phenotype of maize *low phytic acid 1-1* and *low phytic acid 2-1*. *Plant Physiol.* **2000**, *124*, 355–368.
28. Shi, J.; Wang, H.; Wu, Y.; Hazebroek, J.; Meeley, R.; Ertl, D. The maize low-phytic acid mutant *1pa2* is caused by mutation in an inositol phosphate kinase gene. *Plant Physiol.* **2003**, *131*, 507–515.
29. Kim, S.; Tai, T. Identification of novel rice low phytic acid mutations via TILLING by sequencing. *Mol. Breed.* **2014**, *34*, 1717–1729.

30. Kuo, H.; Chang, T.; Chiang, S.; Wang, W.; Charng, Y.; Chiou, T. *Arabidopsis* inositol pentakisphosphate 2-kinase, AtIPK1, is required for growth and modulates phosphate homeostasis at the transcriptional level. *Plant J.* **2014**, *80*, 503–515.
31. Lee, H.; Lee, D.; Cho, H.; Kim, S.; Auh, J.; Pai, H. $InsP_6$-sensitive variants of the Gle1 mRNA export factor rescue growth and fertility defects of the *ipk1 low-phytic-acid* mutation in *Arabidopsis*. *Plant Cell* **2015**, *27*, 417–431.
32. Shukla, S.; VanToai, T.; Pratt, R. Expression and nucleotide sequence of an ins (3) p-1 synthase gene associated with low-phytate kernels in maize (*Zea mays* L.). *J. Agric. Food Chem.* **2004**, *52*, 4565–4570.
33. Ali, N.; Paul, S.; Gayen, D.; Sarkar, S.; Datta, K.; Datta, S. Development of low phytate rice by rnai mediated seed-specific silencing of inositol 1,3,4,5,6-pentakisphosphate 2-kinase gene (*IPK1*). *PLoS ONE* **2013**, *8*, doi:10.1371/journal.pone.0068161.
34. Gaedeke, N.; Klein, M.; Kolukisaoglu, U.; Forestier, C.; Muller, A.; Ansorge, M.; Becker, D.; Mamnun, Y.; Kuchler, K.; Schulz, B.; *et al*. The *Arabidopsis thaliana* ABC transporter AtMRP5 controls root development and stomata movement. *EMBO J.* **2001**, *20*, 1875–1887.
35. Klein, M.; Perfus-Barbeoch, L.; Frelet, A.; Gaedeke, N.; Reinhardt, D.; Mueller-Roeber, B.; Martinoia, E.; Forestier, C. The plant multidrug resistance ABC transporter AtMRP5 is involved in guard cell hormonal signalling and water use. *Plant J.* **2003**, *33*, 119–129.
36. Suh, S.J.; Wang, Y.F.; Frelet, A.; Leonhardt, N.; Klein, M.; Forestier, C.; Mueller-Roeber, B.; Cho, M.H.; Martinoia, E.; Schroeder, J.I. The ATP binding cassette transporter AtMRP5 modulates anion and calcium channel activities in *Arabidopsis* guard cells. *J. Biol. Chem.* **2007**, *282*, 1916–1924.
37. Lee, E.; Kwon, M.; Ko, J.; Yi, H.; Hwang, M.; Chang, S.; Cho, M. Binding of sulfonylurea by AtMRP5, an *Arabidopsis* multidrug resistance-related protein that functions in salt tolerance. *Plant Physiol.* **2004**, *134*, 528–538.
38. Nagy, R.; Grob, H.; Weder, B.; Green, P.; Klein, M.; Frelet-Barrand, A.; Schjoerring, J.; Brearley, C.; Martinoia, E. The *Arabidopsis* ATP-binding cassette protein AtMRP5/AtABCC5 is a high affinity inositol hexakisphosphate transporter involved in guard cell signaling and phytate storage. *J. Biol. Chem.* **2009**, *284*, 33614–33622.
39. Steger, D.; Haswell, E.; Miller, A.; Wente, S.; O'Shea, E. Regulation of chromatin remodeling by inositol polyphosphates. *Science* **2003**, *299*, 114–116.
40. Wilcox, J.; Premachandra, G.; Young, K.; Raboy, V. Isolation of high seed inorganic P, low-phytate soybean mutants. *Crop Sci.* **2000**, *40*, 1601–1605.
41. Oltmans, S.; Fehr, W.; Welke, G.; Raboy, V.; Peterson, K. Agronomic and seed traits of soybean lines with low-phytate phosphorus. *Crop Sci.* **2005**, *45*, 593–598.
42. Hulke, B.; Fehr, W.; Welke, G. Agronomic and seed characteristics of soybean with reduced phytate and palmitate. *Crop Sci.* **2004**, *44*, 2027–2031.
43. Pilu, R.; Panzeri, D.; Cassani, E.; Cerino Badone, F.; Landoni, M.; Nielsen, E. A paramutation phenomenon is involved in the genetics of maize low phytic acid1-241 (*lpa1-241*) trait. *Heredity* **2009**, *102*, 236–245.
44. Panzeri, D.; Cassani, E.; Doria, E.; Tagliabue, G.; Forti, L.; Campion, B.; Bollini, R.; Brearley, C.A.; Pilu, R.; Nielsen, E.; *et al*. A defective ABC transporter of the MRP family, responsible for the

bean lpa1 mutation, affects the regulation of the phytic acid pathway, reduces seed *myo*-inositol and alters aba sensitivity. *New Phytol.* **2011**, *191*, 70–83.
45. Pilu, R.; Panzeri, D.; Gavazzi, G.; Rasmussen, S.; Consonni, G.; Nielsen, E. Phenotypic, genetic and molecular characterization of a maize low phytic acid mutant (*lpa241*). *Theor. Appl. Genet.* **2003**, *107*, 980–987.
46. Pilu, R.; Landoni, M.; Cassani, E.; Doria, E.; Nielsen, E. The maize *lpa241* mutation causes a remarkable variability of expression and some pleiotropic effects. *Crop Sci.* **2005**, *45*, 2096–2105.
47. Cerino Badone, F.; Amelotti, M.; Cassani, E.; Pilu, R. Study of low phytic acid1-7 (*lpa1-7*), a new *ZmMRP4* mutation in maize. *J. Hered.* **2012**, *103*, 598–605.
48. Doria, E.; Galleschi, L.; Calucci, L.; Pinzino, C.; Pilu, R.; Cassani, E.; Nielsen, E. Phytic acid prevents oxidative stress in seeds: Evidence from a maize (*Zea mays* L.) low phytic acid mutant. *J. Exp. Bot*. **2009**, *60*, 967–978.
49. Cerino Badone, F.; Cassani, E.; Landoni, M.; Doria, E.; Panzeri, D.; Lago, C.; Mesiti, F.; Nielsen, E.; Pilu, R. The low phytic acid1-241 (*lpa1-241*) maize mutation alters the accumulation of anthocyanin pigment in the kernel. *Planta* **2010**, *231*, 1189–1199.
50. Landoni, M.; Cerino Badone, F.; Haman, N.; Schiraldi, A.; Fessas, D.; Cesari, V.; Toschi, I.; Cremona, R.; Delogu, C.; Villa, D.; *et al.* Low phytic acid 1 mutation in maize modifies density, starch properties, cations, and fiber contents in the seed. *J. Agric. Food Chem.* **2013**, *61*, 4622–4630.
51. Shi, J.; Wang, H.; Schellin, K.; Li, B.; Faller, M.; Stoop, J.; Meeley, R.; Ertl, D.; Ranch, J.; Glassman, K. Embryo-specific silencing of a transporter reduces phytic acid content of maize and soybean seeds. *Nat. Biotech.* **2007**, *25*, 930–937.
52. Liu, Z.; Cheng, F.; Cheng, W.; Zhang, G. Positional variations in phytic acid and protein content within a panicle of japonica rice. *J. Cereal Sci.* **2005**, *41*, 297–303.
53. Naidoo, R.; Tongoona, P.; Derera, J.; Laing, M.; Watson, G. Combining ability of low phytic acid (*lpa1-1*) and quality protein maize (qpm) lines for seed germination and vigour under stress and non-stress conditions. *Euphytica* **2012**, *185*, 529–541.
54. Xu, X.; Zhao, H.; Liu, Q.; Frank, T.; Engel, K.; An, G.; Shu, Q. Mutations of the multi-drug resistance-associated protein ABC transporter gene 5 result in reduction of phytic acid in rice seeds. *Theor. Appl. Genet.***2009**, *119*, 75–83.
55. Zhang, W.; Gruszewski, H.; Chevone, B.; Nessler, C. An arabidopsis purple acid phosphatase with phytase activity increases foliar ascorbate. *Plant Physiol.* **2008**, *146*, 431–440.
56. Bregitzer, P.; Raboy, V. Effects of four independent low-phytate mutations on barley agronomic performance. *Crop Sci.* **2006**, *46*, 1318–1322.
57. Raboy, V.; Peterson, K.; Jackson, C.; Marshall, J.; Hu, G.; Saneoka, H.; Bregitzer, P. A substantial fraction of barley (*Hordeum vulgare* L.) low phytic acid mutations have little or no effect on yield across diverse production environments. *Plants* **2015**, *4*, 225–239.
58. Karner, U.; Peterbauer, T.; Raboy, V.; Jones, D.; Hedley, C.; Richter, A. Myo-inositol and sucrose concentrations affect the accumulation of raffinose family oligosaccharides in seeds. *J. Exp. Bot.* **2004**, *55*, 1981–1987.
59. Dorsch, J.; Cook, A.; Young, K.; Anderson, J.; Bauman, A.; Volkmann, C.; Murthy, P.; Raboy, V. Seed phosphorus and inositol phosphate phenotype of barley low phytic acid genotypes. *Phytochemistry* **2003**, *62*, 691–706.

60. Ye, H.; Zhang, X.; Broughton, S.; Westcott, S.; Wu, D.; Lance, R.; Li, C. A nonsense mutation in a putative sulphate transporter gene results in low phytic acid in barley. *Funct. Integr. Genom.* **2011**, *11*, 103–110.
61. Rehman, A.; Shunmugam, A.; Arganosa, G.; Bett, K.; Warkentin, T. Inheritance of the low-phytate trait in pea. *Crop Sci.* **2012**, *52*, 1171–1175.
62. Shunmugam, A.S.K.; Bock, C.; Arganosa , G.C.; Georges, F.; Gray, G.R. and Warkentin , T.D. Accumulation of phosphorus-containing compounds in developing seeds of low-phytate pea (*Pisum sativum* L.) mutants. *Plants* **2015**, 4, 1–26.
63. Raboy, V. *Myo*-inositol-1,2,3,4,5,6-hexakisphosphate. *Phytochemistry* **2003**, *64*, 1033–1043.

© 2015 by the authors; licensee MDPI, Basel, Switzerland. This article is an open access article distributed under the terms and conditions of the Creative Commons Attribution license (http://creativecommons.org/licenses/by/4.0/).
